# Did Your Mum Not Hug You Enough? The Effects of Attachment Experience and Callous-Unemotional Traits on Catcalling Behavior in Men

**DOI:** 10.1177/10778012251362216

**Published:** 2025-07-28

**Authors:** Alina Zahn, Florian Hutzler, Sarah Schuster

**Affiliations:** 1 Department of Psychology, Centre for Cognitive Neuroscience, 111602 Paris Lodron University of Salzburg, Salzburg, Austria

**Keywords:** catcalling, street harassment, sexual harassment, parental attachment, callous-Unemotional traits

## Abstract

Catcalling is a type of street harassment which occurs almost daily for most women since their teenage years. The present study aimed to investigate the developmental mechanisms of street harassment in perpetrators. We tested a mediation model in which negative parental attachment experiences are linked to the manifestation of callous-unemotional (CU) Traits which may relate to catcalling. Our model revealed that positive maternal attachment experience is negatively associated with the manifestation of CU Traits, that are further associated with catcalling. Negative paternal attachment experience was directly associated with catcalling. This effect, however, was not modulated via CU Traits. Our findings suggest an inclined position of the maternal relationship quality in the developmental aspects of CU Traits and, in further consequence, street harassment.

## Introduction

### Background

Almost every woman has experienced some form of sexual harassment by the time she turns twenty-four (UN Women National Committee, 2021). A common form of sexual harassment that women are confronted with is *catcalling*—a type of street harassment which occurs almost daily for most women since their teenage years (Johnson & Bennett, 2015; Vera-Gray, 2016). *Street harassment* is a broad term that covers the experience of unwanted sexual attention from strangers in public spaces. The act itself may take a variety of forms. Catcalling covers verbal and non-verbal behaviors such as whistling, pick-up lines, name-calling, leering, winking, gestures commenting on a woman's physical appearance or suggesting sexual activity, touching of one's genitals or even flashing those (Bowman, 1993; Chhun, 2011; Gardner, 1995; Leonardo, 1981; Wesselmann & Kelly, 2010). Touching or stalking is usually not considered as catcalling, but are also forms of severe street harassment.^1^

Catcalling and street harassment in general are widely experienced by women, with around 80% reporting having been harassed by a stranger in a public place (Stop Street Harassment [SSH], 2022). Rates are even higher for transgender and nonbinary people as well as people with disabilities (Kearl, 2014, 2018). However, it is not only women who are victims. Men who report being harassed (prevalence ∼30%; Kearl, 2018) may not rate it as threatening as women do, and - when perpetrated by other men - may not categorize it as street harassment, but rather as an act of racism, transphobia, etc.

Situational and personal factors can lead to catcalling being dismissed as a compliment not only by the perpetrator but also by the victim (Benard & Schlaffer, 1984; Chhun, 2011; Gardner, 1995; Gennaro & Ritschel, 2019; Grossman, 2008; Kissling & Kramarae, 1991; O’Leary & Baldwin, 2016). This can be explained by the fact that catcalling is often used to judge about the sexual attractiveness of the recipient's appearance. However, it has been shown that women feel similar levels of anxiety regardless of the specific context (e.g., irrespective of whether they considered the perpetrator attractive or unattractive). Thus, fear is ever present, even when the catcall is interpreted as a complement (Fairchild, 2010). Regardless of the recipient's interpretation of the perpetrator's intentions, the psychological consequences of catcalling, which is intrusive and objectifying in nature, need to be taken seriously (Vera-Gray, 2016).

The effects of street harassment on the victims are both physical and psychological, ranging from acute distress (e.g., muscle tension, dizziness, nausea, tachycardia, numbness; Tuerkheimer et al., 1997) up to long-term consequences such as increase fear of rape (Fairchild & Rudman, 2008), symptoms of post-traumatic stress disorder (Carretta & Szymanski, 2020), poor quality of sleep (DelGreco & Christensen, 2019) and internalization of negative comments about one's body (Davidson et al., 2015; Fisher et al., 2019). These consequences are risk factors for many psychological disorders, such as depression, anxiety and eating disorders (Ståhl & Dennhag, 2021; UNICEF, 2021).

Men often view catcalling as a compliment that attempts to express their sexual interest in the targeted victim (Walton & Pedersen, 2021). Typical justifications for catcalling include that it is “harmless fun”, alleviates boredom, and facilitates bonding and friendship with other men (Bailey, 2016; Benard & Schlaffer, 1984; Wesselmann & Kelly, 2010). In addition, congruent group norms (Cialdini et al., 1991; Forsyth, 1995; Pryor et al., 1993), group anonymity (Gardner, 1995; Postmes & Spears, 1998), and a constructed “us against them” mentality increase street harassment in men (Dall'Ara & Maass, 1999; Wesselmann & Kelly, 2010). It is worth noting that 15% of the male participants expressed an explicit desire to upset or humiliate women with their catcalls. Furthermore, men who catcalled showed higher levels of hostile sexism, self-described masculinity, social dominance orientation, and tolerance of sexual harassment (Walton & Pedersen, 2021).

The affectively deficient and uncaring attitudes described above are the main features of so-called callous-unemotional (CU) Traits, which are characterized by a lack of guilt and empathy (Essau et al., 2006; Frick et al., 2014; Viding et al., 2014, 2019). Individuals who score higher on these Traits have a higher risk of engaging in antisocial behavior such as humiliating or bullying others, and delinquency in general (Caiozzo et al., 2016; Crass & Terranova, 2018; Golmaryami, Vaughan & Frick, 2021; Loeber, Lacourse, et al., 2005; Loeber, Pardini, et al., 2005). CU Traits can already be observed in childhood and are a precursor of psychopathic traits in adulthood (Longman et al., 2016; Lynam et al., 2007). Longitudinal data indicate that CU Traits may predict serious and persistent criminal behavior in boys, over and above the presence of conduct disorder symptoms and oppositional defiant problems (Pardini & Fite, 2010). In general, boys show higher levels of CU Traits than girls, with a developmental peak at the age of 15 (Essau et al., 2006; Herpers et al., 2012).

Critically, it has been shown that, in addition to genetic predispositions (Mann et al., 2015; Tuvblad et al., 2014; Viding & Larsson, 2007) and neurological alterations (see Herpers et al., 2014, for a review), negative parental attachment experiences are associated with CU Traits and may therefore also be an important risk factor for delinquency (e.g., Bohlin et al., 2012). Ever since Bowlby showed the importance of the bond a child forms with its caregiver for psychological development, there has been consistent support for the idea that attachment style affects almost every part of human life: Securely attached children have higher self-esteem, greater life satisfaction, healthier lifestyles, and more stable friendships and romantic relationships later in life (Bowlby, 1982; Ciocca et al., 2015; Simpson, 1990). In contrast, insecurely attached individuals are more likely to perceive their environment as hostile and find it difficult to form positive social relationships with others. As a result, insecure attachment is associated with externalizing behaviors, substance use disorders, delinquency, antisocial personality traits, and hostile sexism (Bowlby, 1944; Fagot & Kavanagh, 1990; Fearon et al., 2010; Fisher & Hammond, 2019; Schindler & Bröning, 2015).

### The Present Study

The present study aims to investigate the relationship between attachment and CU Traits on street harassment in adult male participants. We hypothesized that representations of parental attachment would be related to catcalling behavior in males and that this relationship would be mediated by lack of empathy and disregard for others, as assessed by CU Traits. Thus, the present study aims to investigate a rather neglected topic in the literature (but see DelGreco et al., 2021), namely the developmental aspects of perpetrators who engage in street harassment. Consistent with previous studies showing reliable associations between CU Traits on the one hand and delinquent behavior (Ansel et al., 2015; Frick et al., 2005; Hawes & Dadds, 2005; Hawes et al., 2014; Viding et al., 2009) or demeaning attitudes (e.g., Bruckner et al., 2024) on the other hand, we suggest that the severity of catcalling behavior is related to the extent of individual manifestations of CU Traits.

Previous studies have shown that secure maternal attachment is an important (protective) factor regarding the manifestation of CU Traits (e.g., Bruckner et al., 2024; Wright et al., 2018). While maternal and paternal attachment quality have been reported to be strongly correlated, attachment security with fathers tends to be lower than with mothers (see Pinquart, 2023, for a recent meta-analysis). The extent to which attachment to the father modulates the association between CU Traits and, ultimately, street harassment is an open question. It is reasonable to assume that, in principle, attachment to the father has similar effects on CU Traits (and consequently on the severity of catcalling behavior) - but that these effects are less pronounced.

## Method

*Participants*. Three hundred sixty-seven male participants from Austria and Germany took part in our online survey, which was implemented in Limesurvey (v.5.6.25). Participants were recruited via social media from February to June 2023. Two hundred five completed the survey. From those, we excluded participants who were under the age of 18 and did not identify as male from further analysis. The final sample consisted of 155 men aged from 19 to 83 (*M* = 33 y, *SD* = 16 y). Demographic characteristics of this sample are presented in Table 1. After completing the survey participants had the opportunity to win one of two 50€. The study was conducted in accordance with the Declaration of Helsinki and approved by our local Ethics Committee.

**Table 1.** Sample Characteristics.

Sexual orientation	Heterosexual: 141	Homosexual: 8	Bisexual: 6	
Highest level of education	Compulsory school: 1	Apprenticeship diploma: 26	A-levels: 57	University degree: 71
	yes	no		
In a relationship	87	68		
Primary caregivers				
Biological mother	152	3		
Biological father	147	8		

*Procedure*. The aim of the study was disguised as investigating flirting behavior instead of catcalling or street harassment to avoid potential social desirability biases. The survey began with the assessment of demographic information, followed by a self-report on parental attachment experiences using the *Parental Attachment Questionnaire* (Lutz et al., 1995), the *Inventory of Callous-Unemotional Traits* (Essau et al., 2006), and, lastly, a *Catcalling Inventory* previously used by DelGreco et al. (2021) which was translated into German. At the end of the survey a short debriefing text revealed the actual aim of the study. Participants were required to give written informed consent for participation and the use of their anonymized data. Only complete protocols were considered for subsequent analysis. The corresponding dataset of this study is available through Austrian NeuroCloud (https://anc.plus.ac.at/) at: https://doi.org/10.60817/RH5S-0J79. The ANC is a research data repository designed for FAIR and trustworthy data management in neuroscience (Hutzler & Himmelstoß, 2024). The dataset is assigned a persistent DOI to ensure long-term findability and citation, and metadata are publicly accessible via the DOI landing page.

### Measures

*Questionnaire on Parental Attachment.* To assess attachment quality, we used the questionnaire on parental attachment (FEB) by Lutz et al. (1995) which is based on the Parental Bonding Instrument (PBI) by Parker et al. (1979). Twenty-five items were presented separately for each parent (e.g., “My mother/father let me wear what I wanted.”). Participants were asked to retrospectively rate their guardians’ behavior in the first sixteen years of life on a four-point Likert scale (1= “does not apply at all” to 4 = “applies very much”). An inverse control item was added to both the mother scale and the father scale. If there was no contact with the biological mother or father, participants had to name a substitute person who was perceived as their male/female caregiver (grandparents, foster parents, etc.). This was the case in 1.94 and 5.16% of the cases of mother and father figure, respectively. Participants were also asked for how many years they lived with each parent.

The FEB attachment is assessed on two bipolar dimensions: *care vs. rejection* and *control vs. autonomy*. Naturally, higher values on the care scale are accompanied by lower values on the rejection scale. The same holds true for control and autonomy. *Care* and *autonomy* are important for positive attachment experiences. Conversely, *rejection* and *control* are usually higher in negative attachment experiences (see Lutz et al., 1995). Internal consistency measured in Cronbach's alpha was 0.90 for both maternal and paternal scales.

*Inventory of Callous – Unemotional Traits.* The German version (Essau et al. 2006) of the Inventory of Callous-Unemotional (CU) Traits by Frick (2004) was used for the present study. The self-report questionnaire consists of 24 items which have to be rated on a four-point Likert scale (1 =  “not at all true” to 4 =  “definitely true”). CU Traits were assessed on three scales: Callousness (i.e., lack of empathy, guilt and remorse for wrongdoing: “I don't care who I hurt to get what I want”), Uncaring (i.e., lack of consideration for one's own performance on tasks and for other people's feelings: “I don't care if I'm on time.”) and Emotionlessness (lack of emotional expression: “I don't show my feelings to others.”). A total score was calculated for the three factors. The internal consistency was in a good range (α = .81).

*Catcalling Inventory.* Catcalling was assessed with a self-report questionnaire by DelGreco and colleagues (2021). Participants were presented with a list of 27 possible behaviors which represent a wide range of street harassment (e.g., “I asked a woman what her name was”, to “I showed a woman my penis in public”). The original version of this questionnaire was developed by Sullivan (2011) and adapted for the perpetrators’ view by DelGreco et al. (2021). The items were translated and back-translated by two independent translators and then compared with the original version. Any discrepancies were discussed and resolved by both translators.

Participants were asked how often they engaged in harassing behavior against women they had never met before in public in the past year (i.e., 0 *=* Never, 1 *=* Once in the past year, 2 *=* A few times in the past year, 3 *=* About once a month, 4 *=* A few times a month, 5 *=* Almost every day, 6 *=* Several times a day). Engaging in each behavior was given a score of one (regardless of how often they actually engaged in the behavior in the past year), resulting in a possible overall street harassment behavior score of 0–27, with higher scores indicating engaging in a greater range of street harassment behaviors.

However, considering only the mere frequency of harassing behavior could lead to somewhat distorted results by neglecting the severity of the behavior in question. In other words, the item “I asked a woman what her name was” could receive the same score as the item “I showed a woman my penis in public”. To address this potential issue, a norming study was conducted to evaluate the severity of each behavior/item from a woman's perspective and to weigh each item in our main study (see Table S1 in the Supplementary Material). The severity score was then multiplied by the frequency of the harassing behavior in question. For the final score, items were summed for each participant. Internal consistency was excellent (α = .96).

### Statistical Analyses

*Descriptives*. Descriptive statistics of the mediation analysis variables can be found in Table 2. A complete overview of the individual items of the catcalling inventory, together with the numbers and percentages of the present sample, is presented in Table 3. As can be seen in Table 3, we observed that every catcalling behavior was indeed represented in our sample. Only 16 men (10%) stated that they did not once engage in any of the listed behaviors within the last year. Of note, we observed no effect of relationship status on catcalling, *t*(151) = -0.12, *p* = .91.

**Table 2.** Descriptive Statistics of the Mediation Analysis Variables.

	*M*	SD	Min	Max
Positive maternal attachment experience	3.13	0.49	1.58	4
Negative maternal attachment experience	1.86	0.49	1	3.24
Positive paternal attachment experience	2.99	0.6	1	4
Negative paternal attachment experience	1.81	0.5	1	3.52
CU traits	45.33	7.72	30	71
Catcalling	65.62	90.48	0	660.76

**Table 3.** Percentage of men Engaging in Different Forms of Street Harassment.

Harassing behavior	*N* (%)
Complimented a woman on her appearance	**115 (74)**
Stared at a woman in a sexual way as you walked past her on the street	**93 (60)**
Asked a woman for her phone number	**69 (45)**
Asked a woman if she had a boyfriend or was married	**66 (43)**
Told a woman to smile	**55 (35)**
Commented on a woman's weight and said she was either too fat or too skinny	**45 (29)**
Made negative comments on a woman's appearance as she walked by	32 (21)
Made gestures and requests to a woman, asking her to come to you	28 (18)
Yelled compliments to a woman about her appearance as she walked past your worksite	15 (10)
Slowed down your car so that you could drive beside a woman as she walked and either watched her or spoke to her	13 (8)
Touched a woman as she walked past you	13 (8)
Walked past a woman and commented on her weight, saying you approve of her size	11 (7)
Told a woman how pretty or attractive she is and then repeated these comments louder to gain her attention	9 (6)
Blown a kiss or made other romantic gestures to a woman on the street	8 (5)
Whistled, yelled or honked at a woman from your car while driving	7 (5)
Approached the male a woman was walking or sitting with and complimented him on her appearance or on his successful conquest	6 (4)
Called a woman insulting names as she walked past you	5 (3)
Yelled things such as “Hey, sexy!” or “You’re fine!” from your car while driving past a woman as she was or waiting for someone	4 (3)
Yelled compliments about a woman's appearance at her while she was jogging	4 (3)
Made sexual comments to a woman and then followed her as she walked away	4 (3)
Made sexually explicit gestures to a woman as she walked by	4 (3)
Walked past and directed sounds at a woman	3 (2)
Called for a woman's attention and when she ignored you, you began shouting insults at	3 (2)
Pulled your car over as a woman was walking and asked her to do sexually explicit things with you	3 (2)
Offered a woman money for sex while she was either walking or waiting for someone	2 (1)
You showed a woman your penis on the street	2 (1)
Aggressively touched a woman as she walked past you	2 (1)

*Note*: Percentages greater than 25% are shown in boldface.

**Figure 1.**
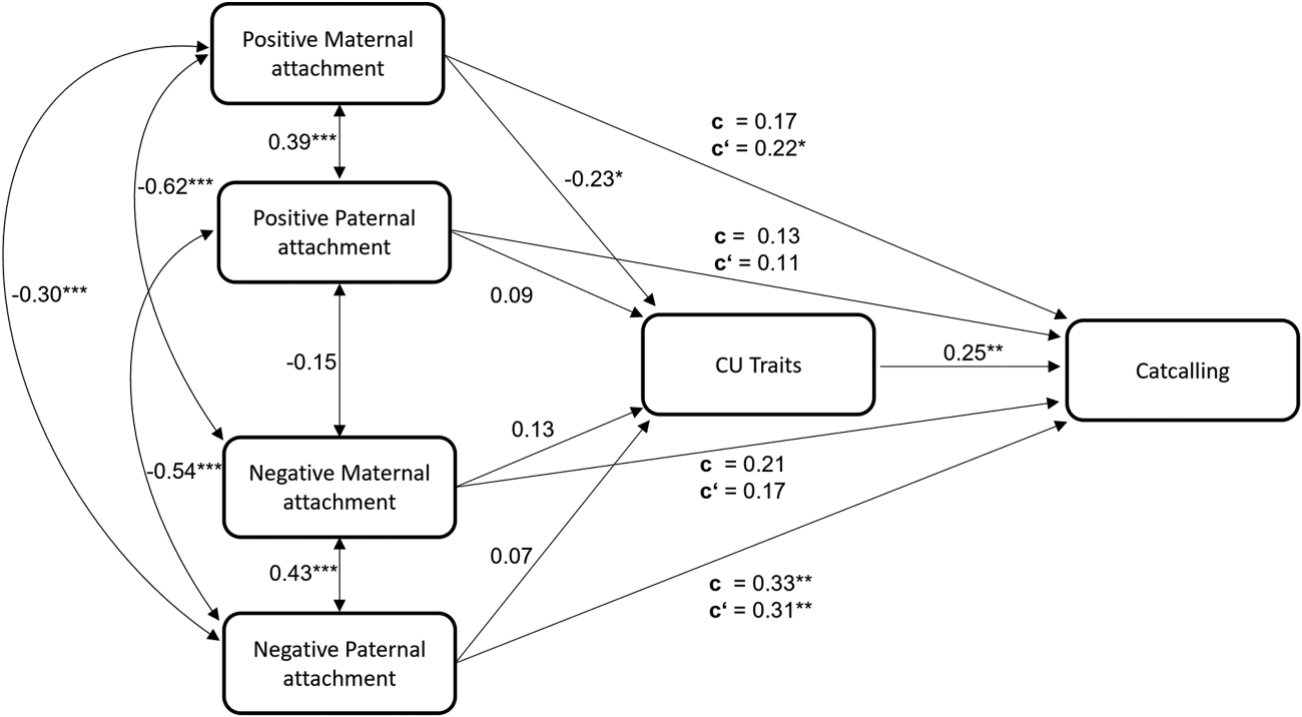
Mediation Model Displaying Effect Estimates among Paths Affecting Catcalling Behavior With Total (c) and Direct Effects (c’). *p < .05; **p < .01; ***p < .001.

*Mediation Analysis.* We performed a mediation analysis to assess the relationship between parental attachment and CU Traits on street harassment. Parental attachment was operationalized by positive and negative attachment experiences in the first sixteen years of life (consisting of the FEB scales *care vs. autonomy* and *rejection vs. control*; see Measures section) with the mother and father, respectively. The structure of the model is presented in Figure 1. Mediation analysis was calculated using the *psych*-package (Revelle, 2022) running in the R environment for statistical computing (R version 3.6.0). Calculation of the confidence intervals of indirect effects was based on bootstrapping with 10,000 replications. Correlation Coefficients between our variables of interest can be found in Table 4.

**Table 4.** Correlation Matrix of the Mediation Analysis Variables.

	1	2	3	4	5	6
1 Positive maternal attachment experience	−	−.62**	.39**	−.30**	−.29**	−.01
2 Negative maternal attachment experience		−	−.15	.43**	.29**	.22*
3 Positive paternal attachment experience			−	−.54**	−.05	−.01
4 Negative paternal attachment experience				−	.14	.30**
5 CU Traits					−	.28**
6 Catcalling						−

*Note*. *p < .01; **p < .001.

## Results

*Mediation Model*. We tested the effect of maternal and paternal attachment experience on catcalling, considering CU Traits as a mediator. As can be seen in Figure 1, CU Traits (controlling for parental attachment) reliably predicted the amount of catcalling behavior, ß = .25, *t*(149) = 3.25, *p* < .01. The effects of parental attachment on catcalling revealed a significant total (c) as well as direct (c’) effect for negative paternal, but not maternal attachment experience, *ß* = .33, *t*(150) = 3.25, *p* < .01; ß = .31, *t*(149) = 3.17, *p* < .01, respectively. Any remaining total effects of the model were not significant, indicating that neither positive or negative maternal nor positive paternal attachment experience by itself could predict catcalling behavior, *t*s(150) < 1.91, *p*s > .06. Positive maternal attachment experience, however, was inversely related to the higher expressions of CU Traits, ß = −.23, *t*(150) = −2.11, *p* < .05, and associated with the amount of catcalling behavior (after accounting for the effect of CU Traits), ß = .22, *t*(149) = 2.13, *p* < .05. We observed no significant indirect effects of parental attachment and CU Traits on catcalling. Notably, using the mere frequency of catcalling behavior as in the original paper by DelGreco and colleagues (2021), yielded very similar results (see Figure S1 in the Supplementary Material).

## Discussion

The present study investigated potential underlying developmental mechanisms for street harassment in perpetrators. We investigated possible associations between the role of maternal and paternal attachment experiences, CU Traits (i.e., lack of guilt and empathy) and catcalling behavior using a mediation model. Catcalling is a subtype of street harassment that occurs almost daily for most women. It refers to the experience of unwanted sexual attention from strangers in public spaces (Johnson & Bennett, 2015; Vera-Gray, 2016) and can vary in its severity from uttering compliments to aggressive sexual advances (see Table 3). Reported negative effects can reach from acute distress (e.g., muscle tension, dizziness, nausea, tachycardia, and numbness; Tuerkheimer et al., 1997) up to long-term consequences, such as heightened fear of rape (Fairchild & Rudman, 2008), symptoms of posttraumatic stress disorder (Carretta & Szymanski, 2020), poor quality of sleep (DelGreco & Christensen, 2019) and internalization of negative comments made about one's body (Davidson et al., 2015) and constitute risk factors for, e.g., depression, anxiety and eating disorders (Ståhl & Dennhag, 2021; UNICEF, 2021). Although street harassment affects all genders, the present study focuses on the male perspective as perpetrator, since women are the recipients of most cases that men initiated (Kearl, 2014).

A first finding of the present study is that only 10% of our current sample of over 150 men reported that they had not engaged in any street harassment in the past year. For the rest of the sample, there was not a single harassing behavior that was not exhibited at least once, showing that catcalling is a societal problem that deserves more attention. The mediation analysis revealed that attachment quality, in terms of maternal and paternal care/rejection and control/autonomy experienced during the first sixteen years of life, was indeed related to catcalling behavior. However, positive and negative paternal and maternal attachment experiences contribute to catcalling in different ways: We observed that higher positive maternal attachment experience (as defined by experienced care and autonomy) is associated with lower manifestation of CU Traits. In turn, higher levels of CU Traits were associated with more and more severe catcalling behavior. In contrast, increased negative paternal attachment experiences (as defined by experienced rejection and control) were directly associated with increased catcalling behavior. This association was not mediated by the manifestation of CU Traits.

The present study's finding that positive maternal attachment experiences are associated with lower manifestations of CU Traits is supported by evidence from the literature (Bisby et al., 2017; Kimonis et al., 2013). Specifically, maternal care is a supposed protective factor in the manifestation of CU Traits (Kyranides et al., 2023; Bisby et al., 2017) and has been associated with increased empathy (Lyons et al., 2017). Empathy has also been shown to mediate between parental attachment experience and forms of delinquency (Thompson & Gullone, 2008), which arguably represents the opposite pole of the CU Trait spectrum. The exact relationship between attachment and CU Traits, however, is still debated with some arguing for a bidirectional relationship between parenting and CU Traits (Hawes et al., 2011). Our findings suggest that maternal relationship quality, compared to paternal relationship quality, plays a dominant role in the development of CU Traits - even though maternal and paternal attachment characteristics are highly correlated (see also Bruckner et al., 2024).

The effect of paternal attachment experience appears to be qualitatively different, with paternal rejection and control being directly related to the severity of catcalling without mediation by CU Traits. Compared to maternal attachment, father-son bonding is thought to be based more on rough and tumble play, which provides a secure base for promoting exploratory behavior (e.g., Grossmann & Grossmann, 2020; Paquette, 2004). Paternal rejection and control experiences during childhood may inhibit this exploration behavior which is assumed to assist in learning how to interact with unfamiliar people and guide their understanding of culture (e.g., Grossmann & Grossmann, 2020). One might further speculate that paternal rejection and control may have been a defining characteristic not only of the relationship with the son, but also of the relationship with the mother figure. Thus, the father's relationship with the mother may have provided an unfavorable role model in dealing/communicating with women. Unfortunately, the fathers’ relationship style with the mother was not assessed in the present study.

An important methodological contribution of the present study is the further development of the catcalling instrument introduced by DelGreco and colleagues (2021). To our knowledge, we are the first to weigh the reported catcalling behavior by its severity as perceived by potential victims. This approach increases the ecological validity of the questionnaires by taking into account that, for example, exposing genitals in front of a victim is a more deviant behavior than making inappropriate comments about a victim's appearance. However, comparing the results of the original analysis strategy and the results of our variant revealed only minor differences, suggesting that once a certain behavioral threshold is crossed, more severe forms of catcalling are very likely to occur. Notably, the most common forms of catcalling in the present sample are consistent with those in the U.S. sample by DelGreco and colleagues (2021).

## Limitations

The present study used self-reports to assess street harassment and retrospectively evaluate parental attachment quality. Social desirability is definitely an issue in answering the present questionnaires due to the sensitive topic. As has been put forward by Walton and Pedersen (2021), participants could have been dishonest due to memory deficits or have misrepresented their engagement in catcalling with some men having genuinely good intentions and have, thus, been falsely categorized. In contrast, some participants may have been intentionally harassing. Thus, as stated by Walton and Pedersen (2021) and Wesselmann and Kelly (2010), results are likely an underestimate of the differences between men who catcall and men who do not, as well as an overestimate of how many men actually have good intentions when getting in touch with women.

Furthermore, the present study was conducted in a cross-sectional design which impedes clear causal inferences regarding the directionality of parental attachment experiences and CU Traits on catcalling behavior. Future longitudinal studies would clearly substantiate the present findings; especially in the light of a bidirectional influence of parenting and CU Traits (van der Zouwen, 2018). Additionally, CU traits can be highly correlated with other significant predictors of conduct problems, such as self-control or sensation seeking (Hadjicharalambous & Fanti, 2018; Mann et al., 2018). Future studies could investigate whether CU Traits independently explain catcalling behavior, or whether their effects could be isolated.

## Conclusions

Our findings suggest that higher attachment quality, in terms of care and autonomy experienced during the first sixteen years of life, is associated with a lower likelihood of street harassment behavior. Specifically, we observed that the experience of positive *maternal* attachment reduced the risk of manifestation of CU Traits. Lower CU Traits, in turn, were associated with lower levels (and severity) of catcalling. Thus, positive care and autonomy experiences during childhood and early adolescence may act as a protective factor in the manifestation of CU Traits and, consequently, delinquent behavior (e.g., Bohlin et al., 2012; Bruckner et al., 2024). Negative paternal attachment experience, as defined by experienced rejection and control, directly predicted catcalling behavior. However, this effect was not mediated by manifestations of CU Traits. Future studies may further explore the differential roles of maternal and paternal attachment experiences in the development of delinquency in general and CU Traits in particular.

## Supplemental Material

sj-docx-1-vaw-10.1177_10778012251362216 - Supplemental material for Did Your Mum Not Hug You Enough? The Effects of Attachment Experience and Callous-Unemotional Traits on Catcalling Behavior in Men Supplemental material, sj-docx-1-vaw-10.1177_10778012251362216 for Did Your Mum Not Hug You Enough? The Effects of Attachment Experience and Callous-Unemotional Traits on Catcalling Behavior in Men by Alina Zahn, Florian Hutzler and Sarah Schuster in Violence Against Women

## Notes

1. Note that we will use the terms catcalling and sexual harassment interchangeably throughout the manuscript.
