# Supplementary material for: Did Your Mum Not Hug You Enough? The Effects of Attachment Experience and Callous-Unemotional Traits on Catcalling Behavior in Men
Source: Violence Against Women. 2025 Jul 28;32(9):3156–73. doi: 10.1177/10778012251362216 (PMC13213034; doi:10.1177/10778012251362216)
Supplement: sj-docx-1-vaw-10.1177_10778012251362216 - Supplemental material for Did Your Mum Not Hug You Enough? The Effects of Attachment Experience and Callous-Unemotional Traits on Catcalling Behavior in Men [file sj-docx-1-vaw-10.1177_10778012251362216.docx]

**Supplementary Material**

**Norming Study**

*Participants.* Ninety adult women completed the severity ratings for the presently used catcalling instrument (*M* = 24 y; *SD* = 7 y). Participants were recruited via social media platforms in Germany and Austria. The survey was conducted online via the LimeSurvey software (Version 5.6.25).

*Procedure*. A brief definition of street harassment/catcalling and the aim of the survey were given at the beginning. The 28 items from the questionnaire (see Table S1) were presented with the instruction to rank (from *1 = not at all* to *10 = extremely*) each item according to the subjective extent of harassing or intruding character of the behavior. The items were rephrased to suit the victim's perspective. Participants were not forced to rank every situation. At the end of the survey, participants had the opportunity to comment or reach out when needed. Means and standard division were calculated and ranked in descending order (see Table 1). Note that for the perpetrator survey, one item is missing (i.e., “*Ask for the name*”) as in the original study of DelGreco et al. (2021).

*Table S1.* Severity ratings (Range: 1-10) of different street harassment behavior.

| Item (translated) | *M* | *SD* |
| --- | --- | --- |
| Compliments your appearance | 4.53 | 2.22 |
| Asks for you number | 5.89 | 2.56 |
| Asks if you have a boyfriend/are married | 6.18 | 2.43 |
| Approaches the male person you are walking or sitting with and complimented him on your appearance or on his successful conquest of you ^a^ | 7.26 | 2.13 |
| Tells you to smile | 7.58 | 2.23 |
| Whistles, yells, or honks at you from his car while you were walking/waiting for the bus/riding bike | 7.60 | 2.20 |
| Walks past you and directed non-verbal sounds at you (catcalls, wolf whistles, etc.) ^a^ | 7.62 | 1.97 |
| Yells compliments to you about your appearance as you walked past their work site (e.g. construction site) | 7.72 | 1.94 |
| Makes gestures and calls for you to come over to where he is standing ^a^ | 7.72 | 2.31 |
| Walks past you and comments on your weight, saying that he approves of your size | 7.83 | 1.89 |
| Stares at you in a sexual way as you are walking past him ^a^ | 7.89 | 2.02 |
| Blows you a kiss on the street or makes romantic gestures | 7.83 | 2.05 |
| Yells things like “hey sexy” or “you‘re fine” from a car while driving past you as you are walking or waiting for someone | 7.98 | 1.83 |
| Makes negative comments about your appearance as you walk by | 8.05 | 2.36 |
| Yells comments about your appearance at you while you are jogging ^a^ | 8.22 | 1.87 |
| Tells you how pretty/attractive you are as you walk down the street and then repeats these comments louder, trying to get your attention? | 8.33 | 1.76 |
| Calls you insulting names to you as you walk past | 8.51 | 2.02 |
| Comments on your weight (e.g. saying you’re too skinny/fat) | 8.55 | 2.00 |
| Calls for your attention and when you ignore him starts insulting you ^a^ | 8.69 | 1.70 |
| Slows down his car so that he can drive beside you as you walk and either watch you or speak to you ^a^ | 9.20 | 1.52 |
| Makes sexually explicit gestures to you as you walk by | 9.26 | 1.49 |
| Touches you as you walk past them (intentionally) ^a^ | 9.39 | 1.57 |
| Offers you money for sex when you are either walking or standing waiting for someone ^a^ | 9.46 | 1.38 |
| Makes sexual comments to you and then follows you as you walk? ^a^ | 9.63 | 1.38 |
| Touches you aggressively when you walk past him ^a^ | 9.67 | 1.40 |
| Pulls his car over as you are walking and asks you to do sexually explicit things with him ^a^ | 9.70 | 1.39 |
| Shows you his penis on the street ^a^ | 9.74 | 1.41 |

*Note*: ^a^ Ratings missing (max. 4 missing values), *M* = Mean; *SD* = Standard Deviation.

*
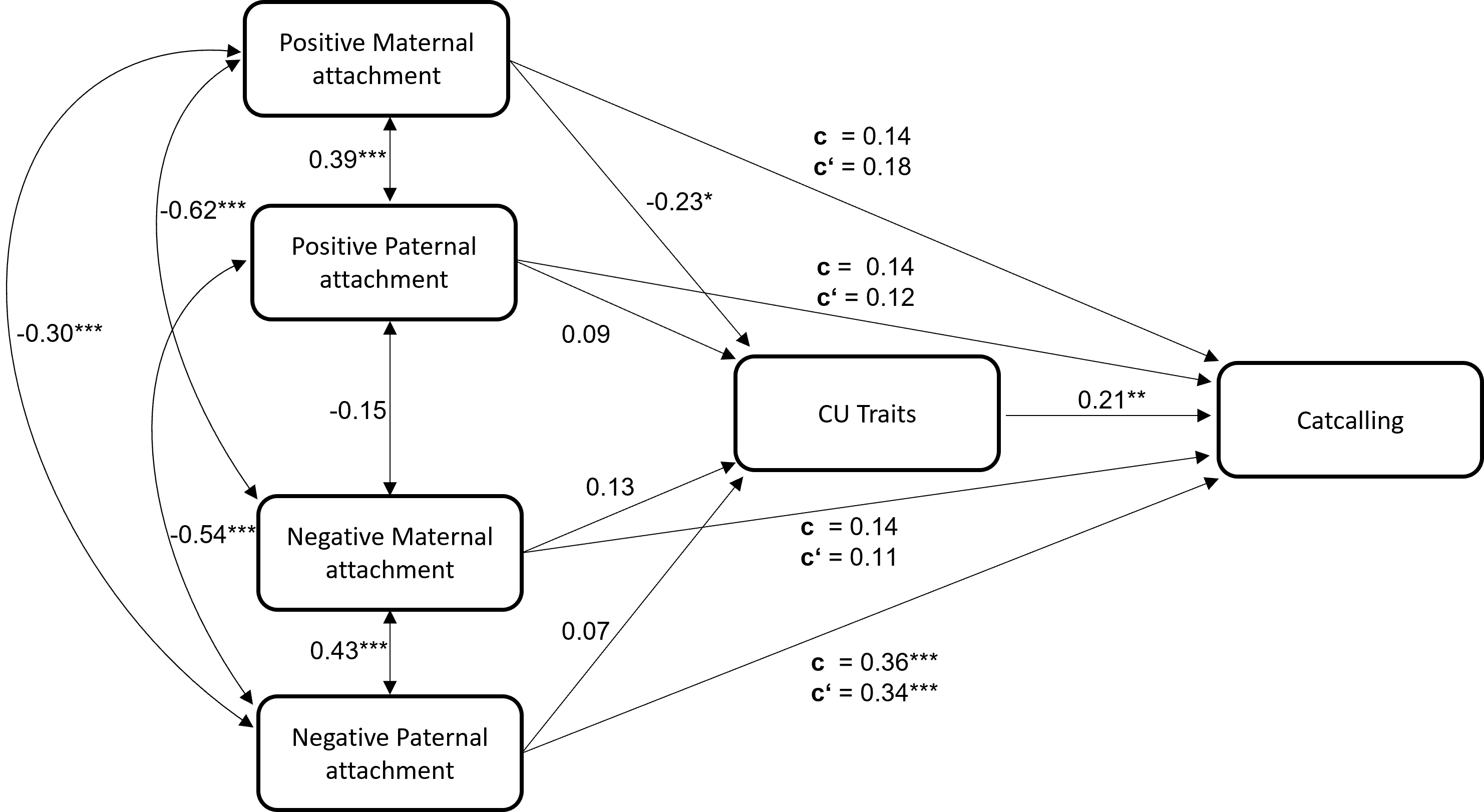
*

*Figure S1*. Mediation model displaying effect estimates among paths affecting catcalling behavior defined according to DelGreco et al. (2021) with total (c) and direct effects (c'). **p* < 0.05; ***p* < 0.01; ****p* < 0.001.
